# A preliminary investigation into the use of molecular oxide and hydride secondary ion relationships for improvement of the ^236^U/^238^U determination on a NanoSIMS 50L

**DOI:** 10.1038/s41598-020-69121-9

**Published:** 2020-07-23

**Authors:** N. Alex Zirakparvar, Cole R. Hexel, Julie B. Smith, Andrew J. Miskowiec, Tyler L. Spano, Roger Kapsimalis

**Affiliations:** Oak Ridge National Laboratory, 1 Bethel Valley Rd, Oak Ridge, TN 37830 USA

**Keywords:** Mass spectrometry, Characterization and analytical techniques

## Abstract

A NanoSIMS 50L is used to investigate uranium molecular (^235^U^16^O, ^236^U^16^O, ^238^U^16^O, ^235^U^1^H, ^238^U^1^H, ^236^U^16^O^1^H, and ^238^U^16^O^1^H) and elemental (^235^U, ^236^U, and ^238^U) secondary ion production during sputtering of synthetic UO_2_ and the NIST-610 standard to determine if: (1) the ^236^U^16^O/^238^U^16^O molecular oxide ratio performs better than the ^236^U/^238^U elemental ratio, and (2) there is co-variance between the molecular hydrides and oxides. Despite an order of magnitude greater abundance of ^236^U^16^O secondary ions (compared to ^236^U), the ^236^U^16^O/^238^U^16^O ratios are less accurate than the ^236^U/^238^U ratios. Further work is needed before the higher count rate of the ^236^U^16^O secondary ion can be used to obtain a better ^236^U/^238^U ratio. The second objective was undertaken because correction for the interference of ^235^U^1^H on the ^236^U secondary ion species typically utilizes the ^238^U^1^H/^238^U ratio. This becomes problematic in samples containing ^239^Pu, so our aim was to understand if the hydride formation rate can be constrained independently of having to measure the ^238^U^1^H. We document correlations between the hydride (^238^U^1^H and ^238^U^16^O^1^H) and oxide (^236^U^16^O) secondary ions, suggesting that pursuing an alternative correction regime is worthwhile.

## Introduction

Secondary ion mass spectrometry (SIMS) is routinely utilized for determining the uranium isotopic composition in a wide variety of materials. For samples containing anthropogenically perturbed uranium, the ^234^U/^238^U, ^235^U/^238^U, and ^236^U/^238^U ratios can be useful indicators of processing history (an up-to-date discussion can be found in^1^). Of these uranium isotope ratios, the ^236^U/^238^U determination by SIMS is particularly challenging for a number of reasons (see discussion by^2^). One reason is that the ‘natural’ ^236^U/^238^U is < 10^–10^^3^, and while the ^236^U/^238^U of anthropogenically modified material can have elevated ^236^U/^238^U, the ^236^U/^238^U ratio is still typically considerably lower than that of the ^235^U/^238^U. This becomes a problem in situations where there is a limited amount of sample available for analysis (e.g. single particle analysis) because precision and accuracy in isotope ratio mass spectrometry are statistically limited by the count-rate of the minor isotope. This is compounded by the fact that the effective transmission of SIMS instruments, which can be thought of as the amount of secondary ions of the target analyte reaching the detector in comparison to the number sputtered from the matrix being analyzed, are typically a few percent at best^4^.

A separate issue that complicates the ^236^U/^238^U determination by SIMS relates to the fact that, when material is sputtered, both elemental and molecular secondary ions are formed. For the ^236^U/^238^U, formation of the ^235^U^1^H hydride molecular species is problematic because a mass resolving power of 38,158 (defined as M/∆M) would be necessary to resolve the ^236^U signal independently of the ^235^U^1^H interference. Such high mass resolving power is not routinely achievable, and even if it were, would come at the expense of secondary ion transmission through the instrument that would, in turn, exacerbate uncertainty introduced in the ^236^U/^238^U ratio by the low ^236^U count-rate. To circumvent this interference, it is possible to monitor the ^238^U^1^H/^238^U ratio, and then apply the hydride formation rate to the observed ^236^U/^238^U using the formula ^236^U/^238^U = ((^236^U + ^235^U^1^H)/^238^U) − (^238^U^1^H/^238^U) × (^235^U/^238^U)^2^ to obtain a corrected ^236^U/^238^U value. While this correction regime has been shown to work for uranium bearing samples that do not contains plutonium, the presence of ^239^Pu within the sample makes the correction regime unusable due to the fact that an MRP of 37,056 is necessary to separate ^238^U^1^H from ^239^Pu.

**Figure 1:**
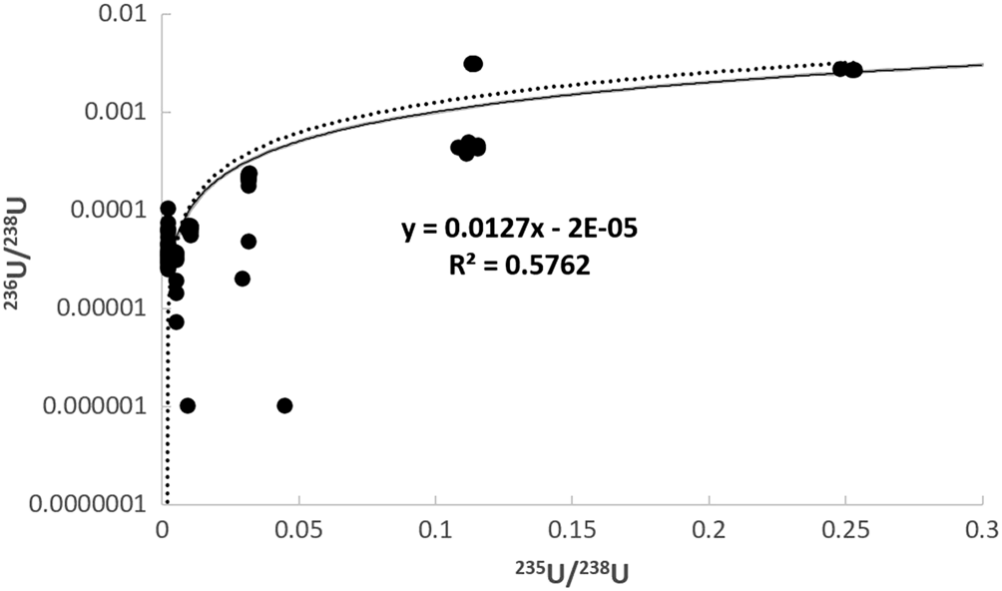
Plot of ^236^U/^238^U versus ^235^U/^238^U ratios determined by SIMS reported in the literature [data compilation can be found in the electronic appendix (supplementary table 3)]. A linear regression through the literature data (the black dotted curve) closely follows the predicted ^235^U^1^H/^238^U ratio at any given ^235^U/^238^U assuming a 1% hydride formation rate (the gray solid curve).

While the ^238^U^1^H/^238^U based hydride correction regime has been shown to work for samples that do not contain Pu, other possible issues relate to the fact that a comparatively high count rate of the ^238^U^1^H is then being applied to the much lower signal at mass ^235^U^1^H + ^236^U by way of the ^235^U/^238^U ratio. In theory, proper detector background and deadtime correction regimes should mitigate any issues related to the drastic differences in count rate between the various secondary ion species, but in practice, these differences could influence the ^236^U/^238^U determination. This is especially true in situations where the ^236^U and ^238^U^1^H are being measured at different times during an analysis (e.g. during magnetic peak hopping) and the molecular hydride formation rate is unstable over the course of that analysis. On Fig. 1, which is a compilation from the literature of reported ^236^U/^238^U ratios plotted as a function of the reported ^235^U/^238^U ratio, it can be seen that there is a broad increase in the reported ^236^U/^238^U ratios with increasing ^235^U/^238^U. Furthermore, this increase (which can be modeled utilizing a power-law curve) broadly follows the hypothetical ^235^U^1^H/^238^U ratios calculated at any given ^235^U/^238^U ratio assuming a 1% hydride formation rate. While there are obviously many nuances specific to the data from each study, the existence of this relationship between the reported ^236^U/^238^U and ^235^U/^238^U ratios suggests that, broadly speaking, the ^236^U/^238^U ratios is not adequately corrected for the ^235^U^1^H interference during SIMS analysis.

The purpose of this study is to investigate two possible avenues for improvement of the ^236^U/^238^U ratio. One possible avenue is in use of the ^236^U^16^O/^238^U^16^O ratio instead of the ^236^U/^238^U ratio. From other studies (e.g.^5^) it is known that the ^23x^U^16^O molecular secondary ions are typically detected in greater abundances as compared to the ^23x^U elemental secondary ions. Therefore, it is possible that the increased count rate of the ^236^U^16^O relative to the ^236^U results in improved precision and accuracy. Use of the ^236^U^16^O molecular ion, as opposed to the ^236^U elemental secondary ion, to determine the abundance of ^236^U, relative to the other isotopes of U in a sample, has not yet been reported in the scientific literature. The second avenue is more exploratory in nature and involves an assessment of whether the pertinent (e.g. ^238^U^1^H for correcting the ^236^U/^238^U or the ^239^U^16^O^1^H for correcting the ^236^U^16^O/^238^U^16^O) hydride formation rates co-vary systematically with the uranium molecular oxide (^238^U^16^O) formation rate. If this co-variability exists, then it may ultimately be possible to constrain the magnitude of the ^235^U^1^H and ^235^U^16^O^1^H interferences on the ^236^U and ^236^U^16^O secondary ions independently of needing to observe the signals at masses ^238^U^1^H and ^238^U^16^O^1^H. Such an approach has not yet been attempted, or even explored, in the scientific literature. These goals are achieved by examination of UO_2_ with a known uranium isotopic composition, as well as the NIST-610 standard whose isotopic composition has previously been documented, on a variety of different substrates and analytical conditions. In the end, we show that both avenues for improvement are viable if a variety of issues can be resolved.

## Results: NanoSIMS data processing and presentation

The raw data from the NanoSIMS, consisting of the total number of counts observed during each of the 6.5 s cycles, was exported from the instrument and processed in Microsoft Excel. The raw data was uncorrected for the electronically programmed 44 ns electron multiplier deadtime, so this correction was applied to the raw data followed by linear interpolation of the secondary ion count rates during each cycle to the time of the magnetic field (B7) containing that cycle’s ^238^U, ^238^U^16^O, and ^238^U^16^O_2_ secondary ion data. This was done to account for any drift in the secondary ion count rates over the course of each cycle. The linearly interpolated and deadtime corrected secondary ion counts were then utilized to calculate the various ratios of interest at each cycle. These cycle-by-cycle ratios were then averaged over the 20 cycles of data collected for each analysis. These ratios are reported in supplementary table 2a and utilized in plots 2 through 9. The error reported for each ratio is the within-run uncertainty on each ratio calculated as standard deviation of the mean value of the 20 cycles of data. In supplementary table 2b, the ratios reported are the weighted mean (calculated using Isoplot v. 3.75^6^) values for individual analyses comprising each of the different substrate and sample types. These weighted mean values were calculated using the within-run 1σ associated with each analysis, but an important note is that the uncertainty reported for these weighted mean values is at the 95% confidence level (2σ). In supplementary table 2b the mean square of weighted deviate (MSWD) value for each of the weighted means is also provided. The values in supplementary table 2a were used to construct all of the figures presented in this study.

## Discussion

The primary goal of this study is to investigate two potential avenues for improvement of the ^236^U/^238^U ratio determination by SIMS. One potential avenue is by use of the ^236^U^16^O/^238^U^16^O ratio instead of the ^236^U/^238^U ratio, which is warranted considering that the uranium oxide (in this case ^235^U^16^O, ^236^U^16^O, and ^238^U^16^O) secondary ions typically exhibit higher count rates than their elemental counterparts (in this case ^235^U, ^236^U, and ^238^U). The second potential avenue of improvement is by searching for evidence of covariance between the ^238^U^1^H and ^238^U^16^O^1^H molecular hydride secondary ion production rates and various other combinations of the uranium elemental and molecular oxide ratios. If such co-variance were to exist, it could mean that it may ultimately be feasible to constrain the uranium hydride secondary ion formation rate taking place during an analysis independently of the assumption that the entire signal at mass 239 is composed entirely of ^238^U^1^H. As discussed in the introduction, the approach of utilizing the ^238^U^1^H/^238^U ratio to correct for the contribution of ^235^U^1^H to the ^236^U/^238^U ratio has been shown to work in samples that do not contain Pu, but this approach cannot work for mixed U–Pu samples. Therefore, consideration of alternative means of constraining the U-hydride secondary ion formation rate during an analysis is a worthwhile endeavor.

**Figure 2:**
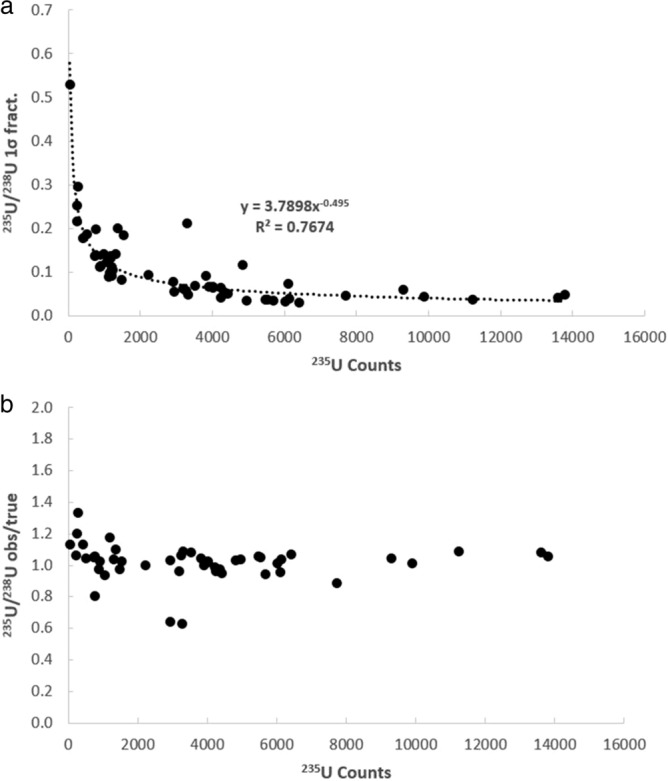
Plots of the fractional 1σ within-run uncertainty (**a**) and observed/true (**b**) versus the total number of ^235^U counts for the ^235^U/^238^U ratio associated with each analysis of the UO_2_ dispersed onto the various substrates.

**Figure 3:**
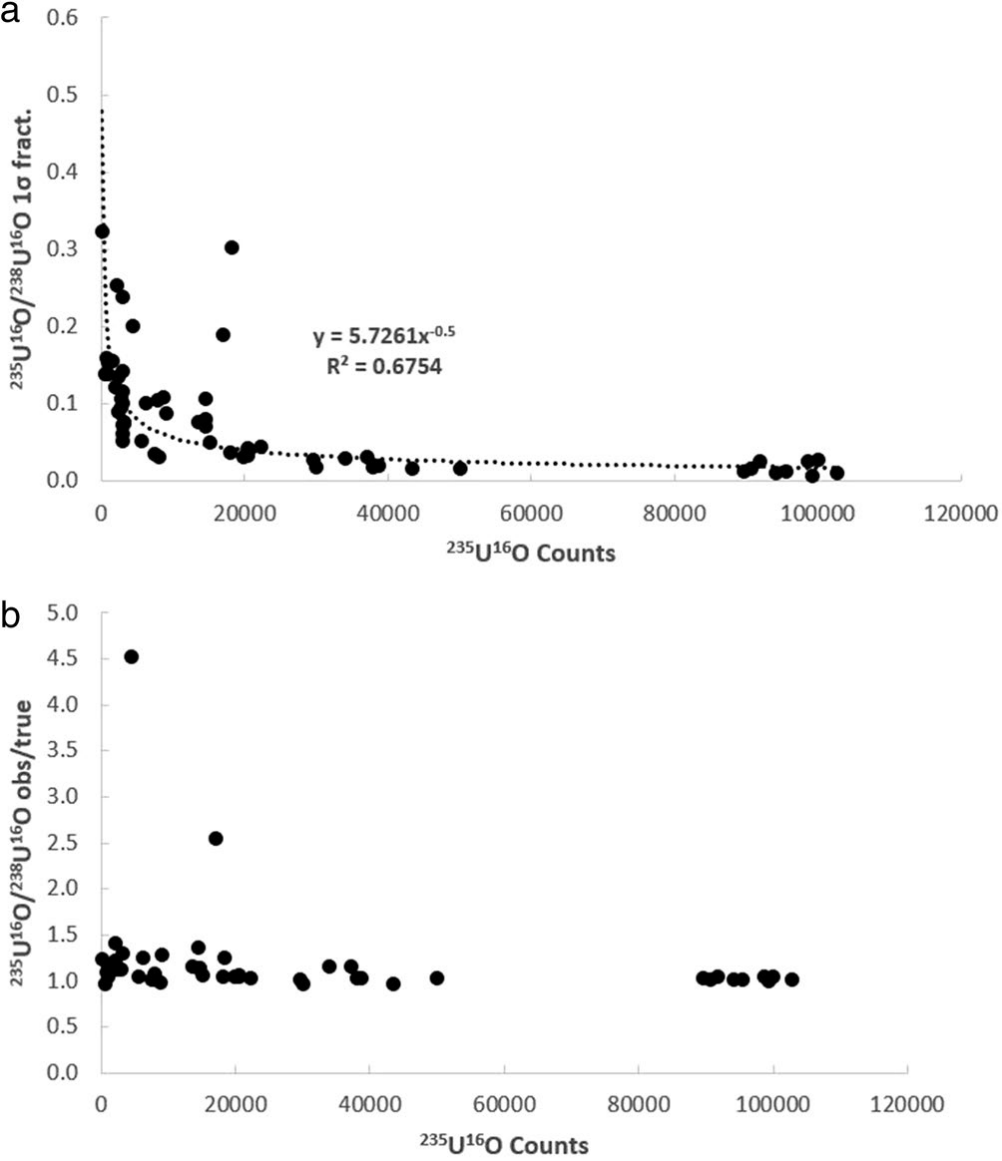
Plots of the fractional 1σ within-run uncertainty (**a**) and observed/true (**b**) versus the total number of ^235^U^16^O counts for the ^235^U^16^O/^238^U^16^O ratio associated with each analysis of the UO2 dispersed onto the various substrates.

**Figure 4:**
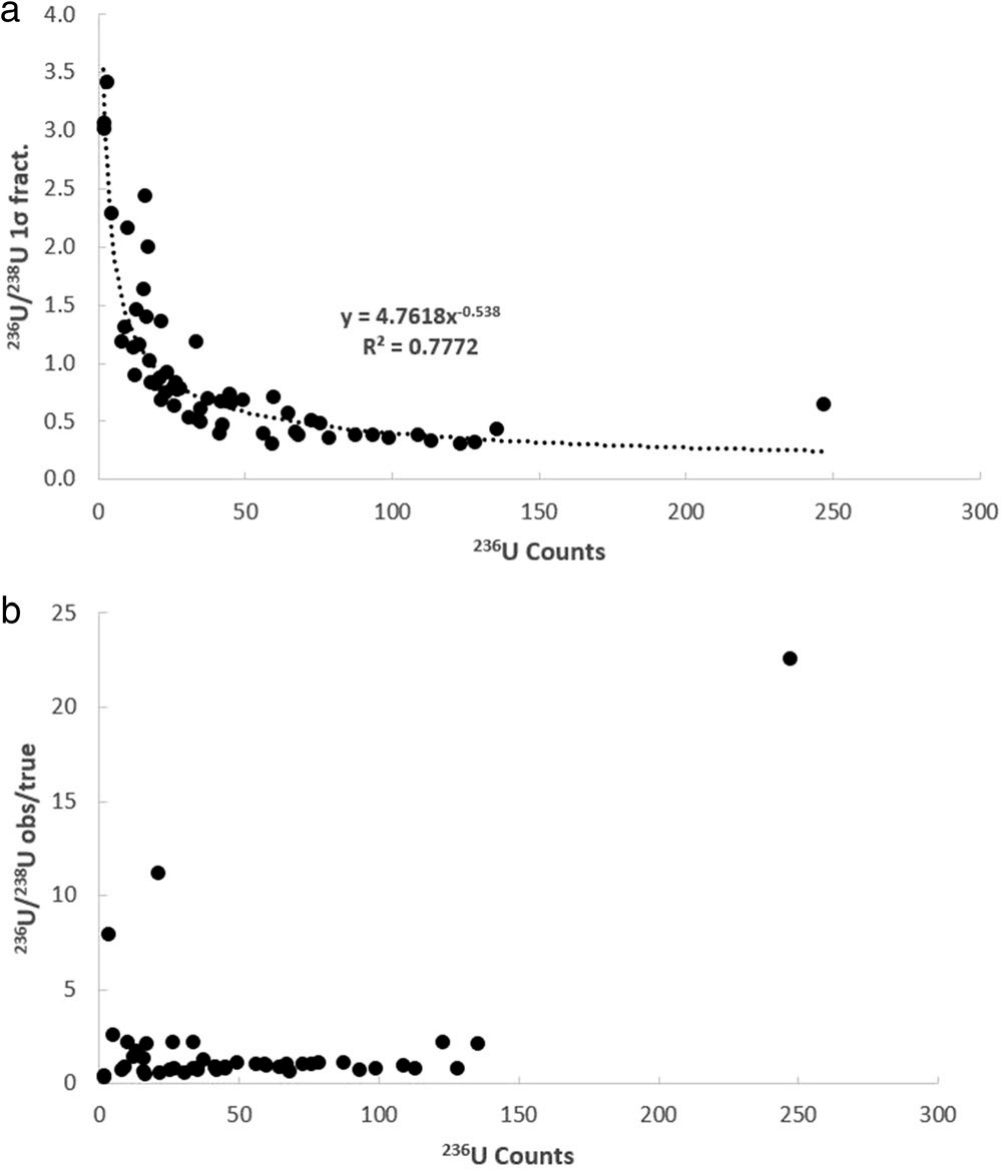
Plots of the fractional 1σ within-run uncertainty (**a**) and observed/true (**b**) versus the total number of ^236^U counts for the ^236^U/^238^U ratio associated with each analysis of the UO_2_ dispersed onto the various substrates.

**Figure 5:**
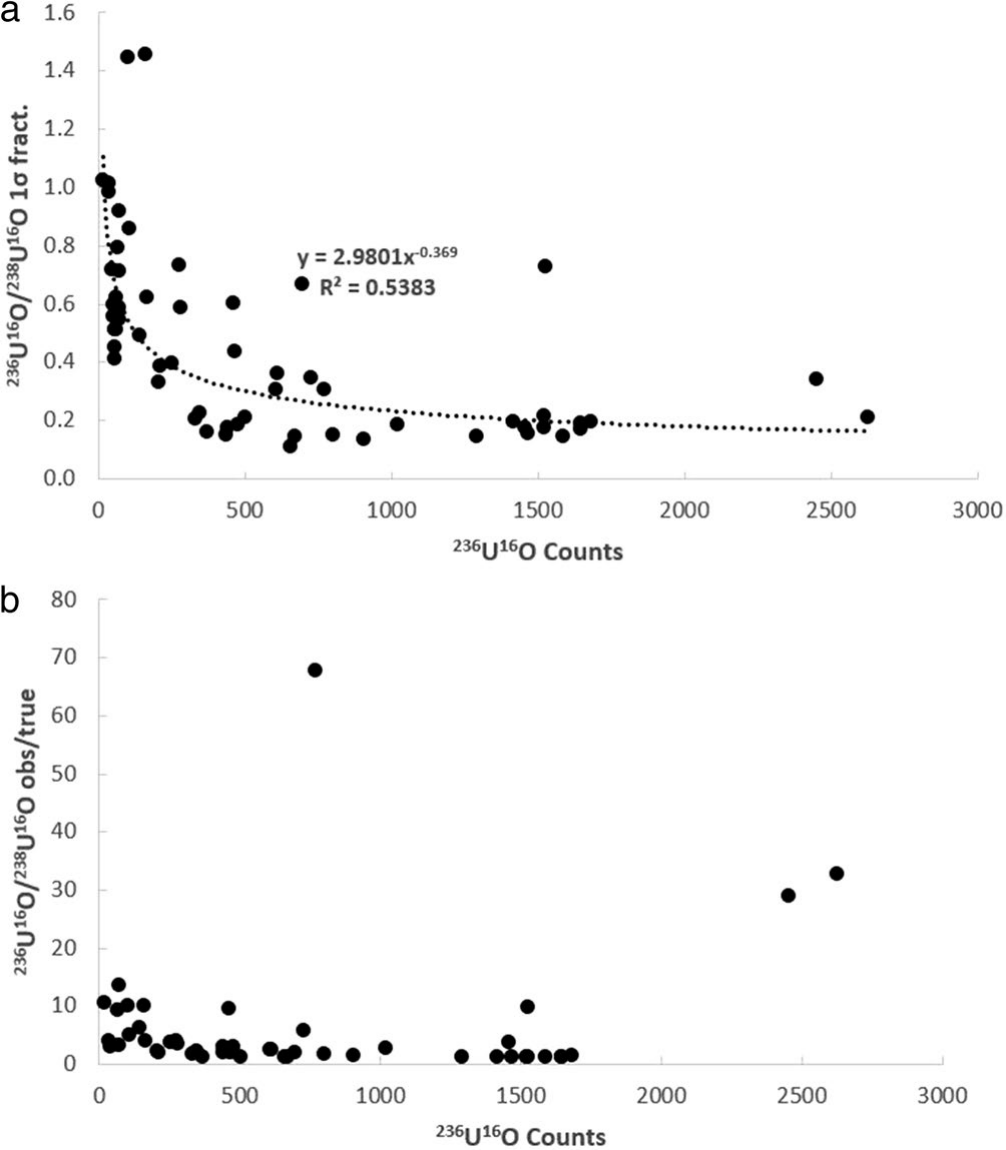
Plots of the fractional 1σ within-run uncertainty (**a**) and observed/true (**b**) versus the total number of ^236^U^16^O counts for the ^236^U^16^O/^238^U^16^O ratio associated with each analysis of the UO_2_ dispersed onto the various substrates.

It is first necessary to consider whether the uranium isotopic data collected in this study is of high enough quality to investigate these possibilities. This can be ascertained on the basis of whether the isotope ratios behave according to the expected relationships between minor isotope count rate, within-run uncertainty, and deviation between the measured and true values. In Fig. 2, the fractional 1σ within-run uncertainty (Fig. 2a) and observed/true (Fig. 2b) versus the total number of ^235^U counts for the ^235^U/^238^U ratio associated are shown for each analysis of the UO_2_ and NIST-610 glass. As can be seen, the within-run 1σ (Fig. 2a) and degree of deviance (Fig. 2b) both decrease exponentially with increasing count rate of the minor isotope (^235^U). The same is true of the ^235^U^16^O/^238^U^16^O within run 1σ (Fig. 3a) and observed/true (Fig. 3b), except that the ^235^U^16^O/^238^U^16^O exhibits a slightly lower within run 1σ, yet a slightly more scattered observed/true, as compared to the ^235^U/^238^U determination. Nonetheless the ^235^U/^238^U and ^235^U^16^O/^238^U^16^O are typically in good agreement with the solution MC-ICP-MS ^235^U/^238^U ratio for the UO_2_ of 0.002153(1) and the published ^235^U/^238^U ratio^7^ for the NIST-610 glass of ^235^U/^238^U = 0.0023856(7). Similar behavior is seen in the uncorrected ^236^U/^238^U (Fig. 4) and ^236^U^16^O/^238^U^16^O ratios (Fig. 5) ratios, as well as the ^234^U/^238^U ratio (not shown in figures, but data is provided in supplementary table 2). The fact that minor isotope count-rate appears to be the major contributing factor to the precision and accuracy of the observed isotopic ratios indicates that the U isotope data collected in this study behaves in-line with what would be expected for mass spectrometric isotope data. Therefore, the dataset can be used to further investigate the two possible avenues for improvement of the ^236^U/^238^U ratio determination outlined in the preceding paragraph.

It is also important to consider the factors impacting hydride formation rate during the analytical session within-which the data presented in this study was collected. ^235^U^1^H hydride formation rate as it applies to the ^236^U/^238U^ determination on micrometer sized particles by SIMS has recently been explored by Simons and Fassett (2017), with a basic observation being that the composition of the analytical substrate upon which the particles are sitting exerts far more control over the hydride formation rate than the residual vacuum within the analytical chamber. The data presented in this study was collected during the course of a single analytical session, wherein the vacuum level within the analysis chamber remained at ~ 4.5 × 10^–9^ mbar. Examination of supplementary table 2a reveals that the ^238^U^1^H/^238^U ratios observed in this study range from as low as 0.00001 to as high as 0.03.

However, it is equally important to note that the ^238^U^1^H/^238^U ratios for the polished UO_2_ and NIST-610 glass reference material are all below 0.0003, whereas the values for the UO_2_ dispersed onto the various substrates are an order of magnitude higher. This could indicate that residual volatiles adhered to the irregular surfaces created by dispersing the finely crushed UO_2_ particles onto the substrates contributed to hydride formation, and/or that the substrates themselves contribute substantially to hydride formation (as suggested by Simons and Fassett, 2017). In either case, obtaining a dataset displaying a range of hydride formation rates is consistent with the goals of our study, which is to explore various hydride correction regimes. An equally important note is that there does not appear to be any statistically significant (e.g. outside of within-run uncertainty) differences in the ^235^U/^238^U or ^235^U^16^O/^238^U^16^O ratios obtained for the highly polished UO_2_ and the UO_2_ that was crushed and dispersed onto the various substrates.

### Use of the ^236^U^16^O/^238^U^16^O vs the ^236^U/^238^U ratio

Comparison between Figs. 4a and 5a reveals that the ^236^U^16^O/^238^U^16^O within-run uncertainties are lower than the ^236^U/^238^U uncertainties, whereas comparison between Figs. 4b and 5b reveals that the raw ^236^U^16^O/^238^U^16^O ratios exhibit more scatter relative to the solution MC-ICP-MS value as compared to the raw ^236^U/^238^U ratios. However, it is important to note that both the ^236^U/^238^U and ^236^U^16^O/^238^U^16^O ratio must be corrected for the ^235^U^1^H and ^235^U^16^O^1^H interferences. For the ^236^U/^238^U, this is achieved by use the formula ^236^U/^238^U = ((^236^U + ^235^U^1^H)/^238^U) − (^238^U^1^H/^238^U) x (^235^U/^238^U) (see “Discussion”^2^). Applying this calculation to the raw ^236^U/^238^U ratios pulls the data from the greased substrates towards lower values that are, on average, closer to the solution value (supplementary table 2b). In contrast, the data from the un-greased substrates are pulled, on average, towards values lower than the solution value. In some cases, the corrected ^236^U/^238^U ratios are ≤ 0. For the polished UO_2_ mounted in epoxy, the correction regime has virtually no effect on the ^236^U/^238^U whereas for the NIST-610 glass, the uncorrected ^236^U/^238^U are actually closer, on average, to the published value than the corrected ^236^U/^238^U (supplementary table 2b) which are pulled towards lower values. These observations suggest that the ^235^U^1^H correction based on the ^238^U^1^H signal results in a slight over-correction of the ^236^U/^238^U ratio for the data set collected in this study.

There are several possible reasons for this. One may be that the hydride formation rate at the time of the ^238^U^1^H acquisition is higher than that at the ^236^U acquisition. Examination of the secondary ion count rate data does indicate that for some of the analyses (full dataset provided in the electronic appendix), the ^238^U^1^H does increase as a function of time during the analytical session whereas in others, it remains stable or decreases. This variability in the count-rate as a function of time during the analysis is the reason why the raw secondary ion count-rates were linearly interpolated to the time of the ^238^U acquisition prior to additional data processing. However, the reality is that the very low ^236^U count rate (sometimes < 1 c/s) complicates the linear interpolation method. Equally problematic is the fact that the hydride formation rate, as constrained by monitoring the relatively strong signal at mass ^238^U^1^H, must then applied to the considerably lower signal at mass ^235^U to infer the number of ^235^U^1^H ions being produced at the time of the ^236^U acquisition. While other factors may be important, the ^236^U/^238^U ratio overcorrection is most likely related to the large difference between the count rates at masses 235, 236, and 239. This is especially true for those analyses where the ^238^U^1^H increases as a function of time whereas, the ^236^U and ^235^U count rates are too low to exhibit a noticeable increase or decrease as a function time during any of the analyses.

While the ^236^U count rate is sometimes < 1 c/s for the analyses conducted in this study, the ^236^U^16^O count rates were typically > 10 c/s. This higher count rate results in a smaller within-run 1σ (Fig. 4a vs 5a), but as noted earlier the raw ^236^U^16^O/^238^U^16^O ratios exhibit more scatter as compared to the raw ^236^U/^238^U ratios (Fig. 4b vs 5b). As with the ^236^U/^238^U ratio, it is necessary to account for an interference on the ^236^U^16^O signal from the ^235^U^16^O^1^H molecular species, which would require a mass resolving power of 40,743 to resolve. Since the dynamics of forming a secondary ion molecular species containing uranium, oxygen, and hydrogen (e.g. ^235^U^16^O^1^H) would presumably be different as compared to one containing only uranium and hydrogen (e.g. ^235^U^1^H) it would be inappropriate to use the observed ^235^U/^238^U and ^238^U^1^H/^238^U to correct the observed ^236^U^16^O/^238^U^16^O ratio. Because the mass table utilized in this study (Table 1) also included the ^235^U^16^O, ^238^U^16^O, and ^238^U^16^O^1^H molecular oxide and hydride species, it is possible to apply a correction according to the following equation: ^236^U^16^O/^238^U^16^O = ((^236^U^16^O + ^235^U^16^O^1^H)/^238^U^16^O) − (^238^U^16^O^1^H/^238^U^16^O) × (^235^U^16^O/^238^U^16^O). In principal this is similar to the equation for the elemental ^236^U/^238^U correction, except that it utilizes the molecular secondary ion species which is likely to be more appropriate for the ^236^U^16^O/^238^U^16^O corrected ratio. Applying this correction scheme does not reduce, on average, the degree of scatter exhibited by the ^236^U^16^O/^238^U^16^O ratios (supplementary table 2b) for the UO_2_ dispersed onto the various substrates. However, for UO_2_ mounted on the carbon sticky tab and mounted in epoxy, as well as the NIST-610 glass, the corrected ^236^U^16^O/^238^U^16^O values do move slightly closer to the ‘true’ values in comparison to the uncorrected ^236^U^16^O/^238^U^16^O ratios.

**Table 1:** NanoSIMS Analytical conditions utilized in this study. All analyses of UO_2_ were conducted with a 200 pA O^-^primary beam rastered over a 5 × 5 µm area. In contrast, the NIST-610 glass was analyzed using a scanning 2 nA primary beam. All analyses consisted of 6.5 s per cycle at each mass station, with a total of 20 cycles of data for each analysis.

Field	EM5	EM6	EM7
B1	Settling field		
B2	^234^U		
B3	^235^U		
B4		^235^U^16^O	
B5	^236^U		
B6		^236^U^16^O	
B7	^238^U	^238^U^16^O	^238^U^16^O_2_
B8	^238^U^1^H	^238^U^16^O^1^H	

The cause of this behavior is not known, but an important observation may be that the ^238^U^16^O^1^H signal exhibits similar behavior to the ^238^U^1^H as a function time within the analysis in that, for some analyses, the signal remains relatively stable whereas in others it increases or decreases throughout the analysis. As with the ^236^U/^238^U correction, the discrepancy between the time of data collection for the ^236^U^16^O and ^238^U^16^O^1^H secondary ion species may lead to some instability in the correction regime that is not adequately accounted for using the linear interpolation method to correct for signal drift. While the uncorrected and corrected ^236^U^16^O/^238^U^16^O ratios do not result in a systematic improvement in their accuracy when compared to the corrected and uncorrected ^236^U/^238^U ratios, the basic observation that the ^236^U^16^O/^238^U^16^O ratios are associated with better within-run precision is promising. If the sources of uncertainty introduced by the hydride correction regime can be resolved, it may ultimately be feasible to take advantage of the improved ^236^U^16^O count rate. However, this will require a better understanding of the molecular oxide and hydride formation dynamics, which will be discussed in the subsequent section.

### Evidence for coupling between the U molecular hydride and oxide secondary ions

As discussed in the preceding section, developing a better understanding of molecular oxide and hydride secondary ion formation may lead to an alternative (i.e. one that is not based on the ^238^U^1^H/^238^U or ^238^U^16^O^1^H/^238^U^16^O ratios) correction regimes for the ^236^U/^238^U ratio determination if it can be shown that the molecular hydride production rate co-varies systematically with the production rate of a non-hydride (and interference free) molecular oxide. It may also help clarify our understanding of how us of the relatively strong signal at mass 239 to correct the comparatively lower signal at mass 236 contributes to uncertainty in the final corrected ^236^U/^238^U ratios. A different situation altogether is where the material under investigation contains both U and Pu. In such an instance, having an alternative correction is especially important since ^238^U^1^H and ^239^Pu would require an MRP of 37,057 to resolve from one another. In this situation, utilizing the ^236^U^16^O/^238^U^16^O ratio would not necessarily ameliorate the issue due to the fact that it would still be necessary to correct for the contribution of ^235^U^16^O^1^H on the observed ^236^U^16^O signal. To do this, one could monitor the ^238^U^16^O^1^H signal (as was done in this study). However, if ^239^Pu is present and is forming ^239^Pu^16^O molecular oxide, which would require an MRP of 39,536 to resolve from the ^238^U^16^O^1^H, it would still not be possible to accurately constrain the hydride formation rate for correction of the ^236^U^16^O/^238^U^16^O ratio for the ^235^U^16^O^1^H interference.

This discussion will now seek to understand whether the secondary ion molecular U hydride formation rate can be constrained independently of the signal at mass 239 (^238^U^1^H) or 255 (^238^U^16^O^1^H). While there is still considerable uncertainty in the SIMS community over the mechanisms governing molecular secondary ion formation, co-variance between different elemental and molecular oxide species has been documented and is actually the basis for certain inter-element correction regimes utilized in other disciplines (see discussion by^8^). Therefore it is possible that there will be some co-variability between either the ^238^U^1^H/^238^U and/or ^238^U^16^O^1^H/^238^U^16^O hydride ratios and a non-hydride molecular oxide secondary ion species (e.g. the ^238^U^16^O/^238^U). If this co-variability exists, it might mean that the formation rate of the non-hydride molecular oxide observed during an analysis could be utilized to infer the hydride formation rate, thus by-passing the need to use the formula ^236^U/^238^U = ((^236^U + ^235^U^1^H)/^238^U) − (^238^U^1^H/^238^U) × (^235^U/^238^U) or its modified version for the ^236^U^16^O/^238^U^16^O ratio correction. In practice, such an approach would obviously require the existence of matrix matched reference materials as well as some other analytical considerations. However, a first step is simply to examine whether there is any evidence of this co-variability. The dataset collected in this study allows us to answer this question since the UO_2_ was examined across a range of different substrates and preparation routes that presumably influence both the hydride and non-hydride molecular secondary ion formation rates. Therefore, if co-variability exists, it should be evident in the dataset collected in this study.

**Figure 6:**
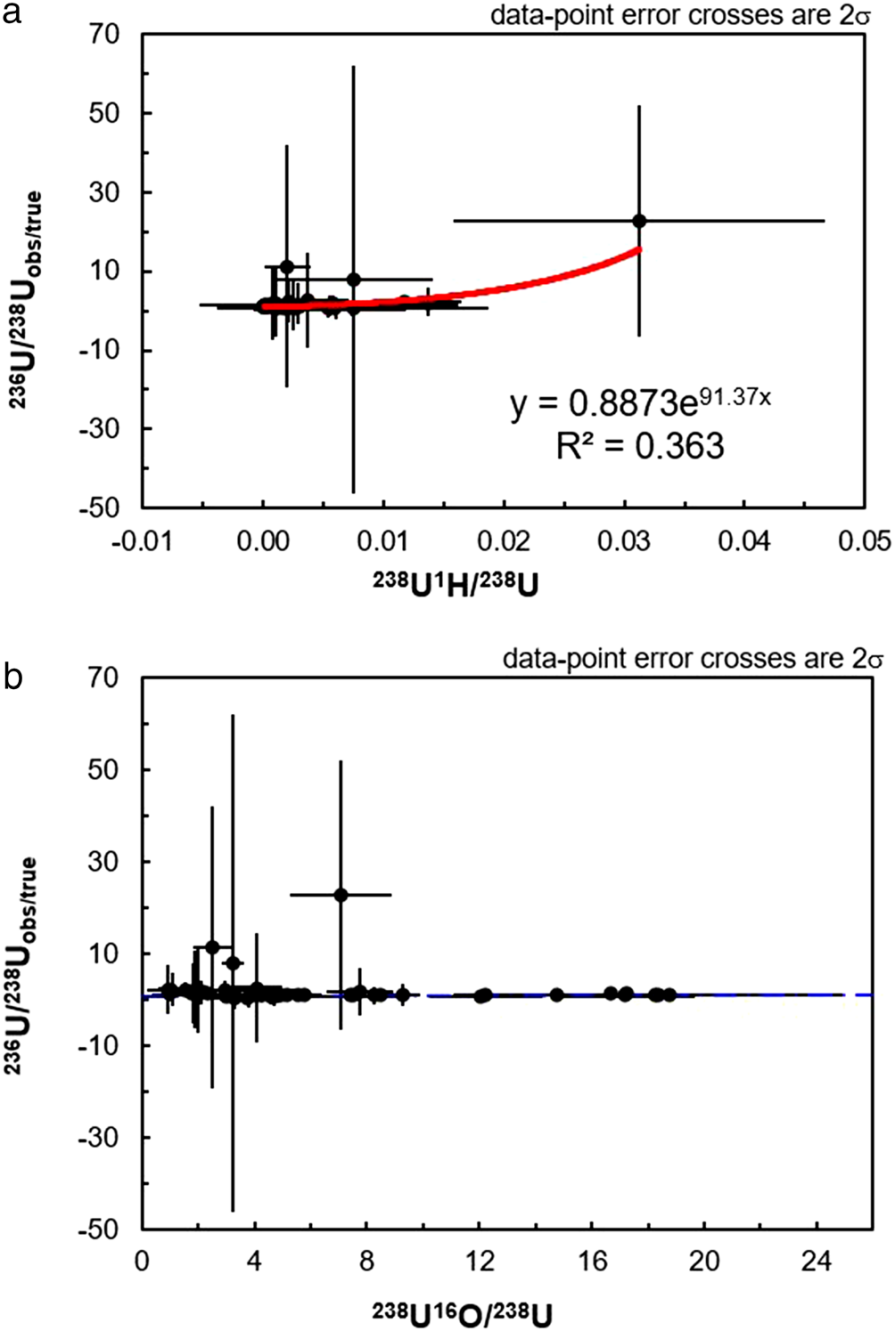
Plots of the ^236^U/^238^U observed/true (and associated 2σ uncertainties) versus the observed ^238^U^1^H/^238^U (**a**) and ^238^U^16^O/^238^U (**b**) ratios.

In Fig. 6a, it can be seen that there is a weak positive correlation between the uncorrected ^236^U/^238^U and the observed ^238^U^1^H/^238^U ratio. However, the vast majority of the datapoints are clustered towards lower ^238^U^1^H/^238^U ratios, whereas the data points with higher ^238^U^1^H/^238^U are also those with the highest levels of within-run uncertainty on the uncorrected ^236^U/^238^U. The lack of a strong positive correlation between the uncorrected ^236^U/^238^U and the observed ^238^U^1^H/^238^U ratio is not surprising given the observations made in the preceding section, that correction of the ^236^U/^238^U using the ^238^U^1^H/^238^U ratio did not uniformly improve the ^236^U/^238^U determination. Unsurprisingly, in Fig. 6b there is no positive correlation between the uncorrected ^236^U/^238^U ratios and the observed ^238^U^16^O/^238^U ratio. As discussed earlier, it is likely that scatter in the ^236^U/^238^U ratios induced by the low count rate of the ^236^U secondary ion supersedes any relationships that may exist between the ^235^U^1^H and ^238^U^1^H hydrides (which should be one-to-one). It is also unclear whether the magnitude of the hydride interference induced shift in the uncorrected ^236^U/^238^U ratios, away from their true values, co-varies with the rate of formation of a non-hydride molecular oxide secondary ion (Fig. 6b).

**Figure 7:**
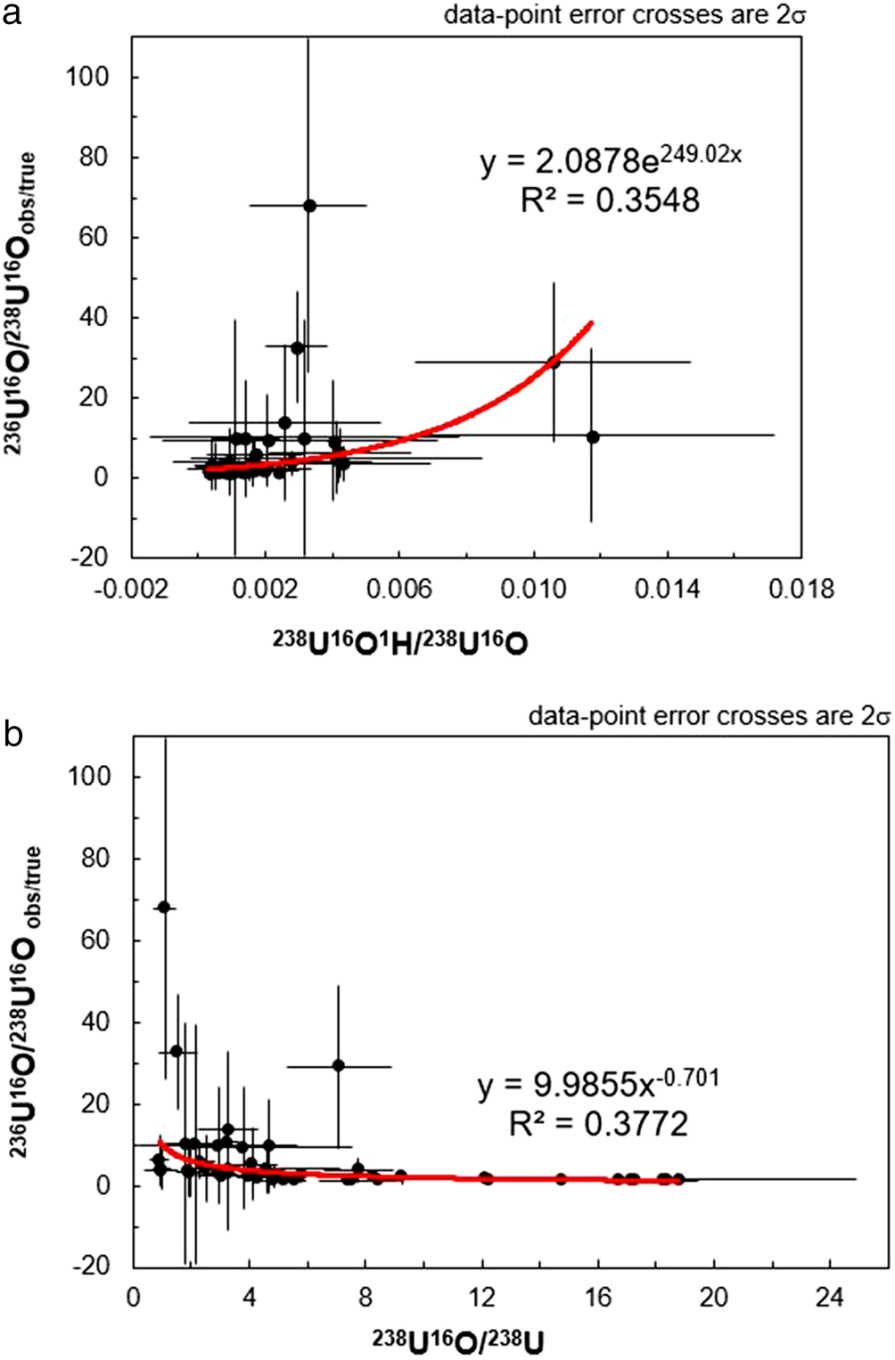
Plots of the ^236^U^16^O/^238^U^16^O observed/true (and associated 2σ) versus the ^238^U^16^O^1^H/^238^U^16^O (**a**) and ^238^U^16^O/^238^U (**b**) ratios.

**Figure 8:**
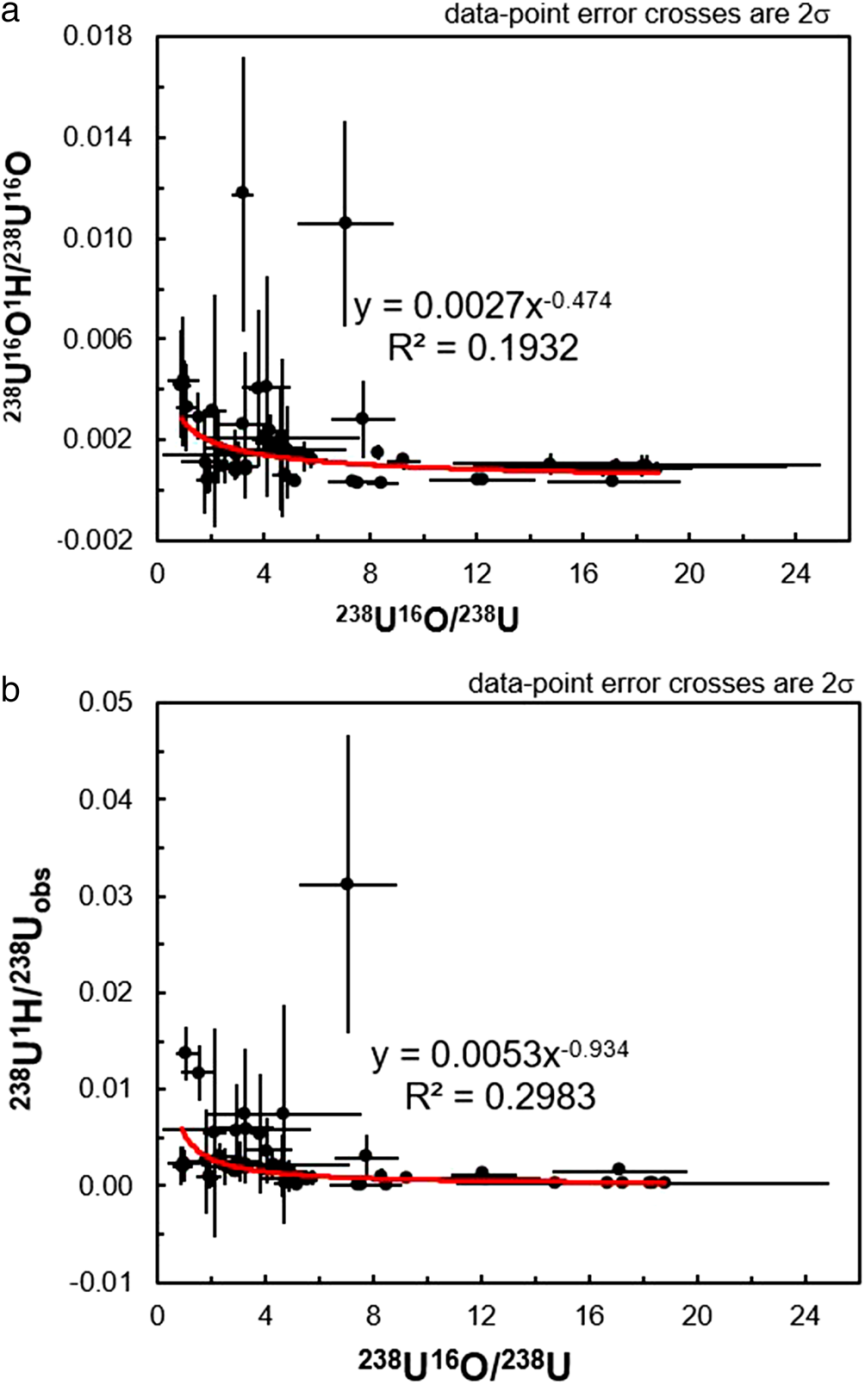
Plots of the ^238^U^16^O^1^H/^238^U^16^O (**a**) and ^238^U^1^H/^238^U (**b**) ratios versus the ^238^U^16^O/^238^U ratio.

In contrast to the lack of any correlations between the ^236^U/^238^U and the observed ^238^U^1^H/^238^U or ^238^U^16^O/^238^U ratios observed on Fig. 6, there does appear to be a stronger relationship between the uncorrected ^236^U^16^O/^238^U^16^O ratios and the ^238^U^16^O^1^H/^238^U^16^O (Fig. 7a) and ^238^U^16^O/^238^U ratios (Fig. 7b). On Fig. 7a, it can be seen that the uncorrected ^236^U^16^O/^238^U^16^O ratios are skewed towards higher values with increasing ^238^U^16^O^1^H/^238^U^16^O, which is the expected behavior. It is therefore unclear why the application of a correction regime based on the ^238^U^16^O^1^H/^238^U^16^O and ^235^U^16^O/^238^U^16^O ratios results in corrected ^236^U^16^O/^238^U^16^O ratios that are more deviated from the solution ^236^U/^238^U value of 0.00002576(4) than the corrected ^236^U/^238^U ratios. In supplementary table 2b, it can be seen that the corrected ^235^U^16^O/^238^U^16^O ratios are actually associated with higher uncertainty and more scatter than the ^235^U/^238^U ratios despite the higher count rate of the ^235^U^16^O secondary ion in comparison to the ^235^U secondary ion. Therefore, one possibility is that this higher degree of scatter in the ^235^U^16^O/^238^U^16^O translates into scatter in the final corrected ^236^U^16^O/^238^U^16^O when using the formula ^236^U^16^O/^238^U^16^O = ((^236^U^16^O + ^235^U^16^O^1^H)/^238^U^16^O) − (^238^U^16^O^1^H/^238^U^16^O) × (^235^U^16^O/^238^U^16^O). While the uncorrected ^236^U/^238^U ratios do not vary systematically with a non-hydride molecular species, examination of Fig. 7b reveals that the uncorrected ^236^U^16^O/^238^U^16^O do vary with the ^238^U^16^O/^238^U ratio. This implies that formation of the hydrogen-bearing molecular oxide secondary ion is at the expense of the ^238^U^16^O molecular oxide, which is confirmed by examination of Fig. 8a where it can be seen that the ^238^U^16^O^1^H/^238^U^16^O ratio deceases with increasing amounts of the ^238^U^16^O molecular species compared to the ^238^U elemental secondary ion. Similar behavior is observed for the ^238^U^1^H/^238^U on Fig. 8b, where it can be seen that the ^238^U^1^H molecular species decreases with increasing ^238^U^16^O production.

The major impetus for exploring an alternate correction regime that does not rely on the direct measurement of the ^238^U^1^H secondary ion species is that the commonly used correction regime relying on the equation ^236^U/^238^U = ((^236^U + ^235^U^1^H)/^238^U) − (^238^U^1^H/^238^U) × (^235^U/^238^U) cannot be used in a situation where the sample contains ^239^Pu. A secondary goal is to assess whether it may be possible to mitigate scatter in the corrected ^236^U/^238^U and ^236^U^16^O/^238^U^16^O ratios that arises from the low count rates of the ^236^U and ^238^U^16^O secondary ion species as well as the magnification of uncertainty that arises when the ^235^U/^238^U and/or ^235^U^16^O/^238^U^16^O is used to apply the hydride formation rate determined by the ^238^U^1^H/^238^U and/or ^238^U^16^O^1^H/^238^U^16^O ratios to the signals at mass ^236^U and/or ^236^U^16^O. Both goals require a practical assessment of how the various secondary ion species behave. As explained above, molecular oxide and hydride production rates do appear coupled. In theory, this coupling could be applied to the analysis of an unknown by taking an approach similar to the reference materials based calibration curves that are widely utilized in SIMS. For example, in considering that the ^238^U^1^H and ^238^U^16^O^1^H production rates are coupled to the UO/U ratio, one approach might be to establish this relationship using standards of known ^236^U/^238^U and that are matrix matched to the unknown in question, and then use the curve to apply a correction to the observed ^236^U/^238^U or ^236^U^16^O/^238^U^16^O during an analysis of the unknown based on that analyses’ observed ^238^U^16^O/^238^U ratio. The dataset does not currently exist to further evaluate such an approach. Application of a correction regime derived from the UO_2_ dataset cannot be appropriately applied to the NIST-610 measurements since these were made using a different primary beam condition. However, the observations made in this study certainly give promise to the possibility that a correction regime for hydride formation independently of having to measure the ^238^U^1^H species is worth pursuing.

## Conclusions

Consideration of the NanoSIMS data for the ^235^U, ^236^U, and ^238^U elemental, the ^235^U^16^O, ^236^U^16^O, and ^238^U^16^O molecular oxide, as well as the ^238^U^1^H and ^238^U^16^O^1^H molecular hydride secondary ions for UO_2_ of known isotopic composition mounted in a variety of forms as well as the NIST-610 glass reference material supports the following conclusions:

1. The higher count rate of the ^236^U^16^O, in comparison to the ^236^U, results in ^236^U^16^O/^238^U^16^O ratios with lower within-run uncertainties in comparison to the ^236^U/^238^U ratios. However, the ^236^U^16^O/^238^U^16^O ratios are more scattered than the ^236^U/^238^U ratios. Therefore, additional work is necessary to identify the cause(s) of this scatter and thus take advantage of the higher count rates afforded by the molecular oxide secondary ion species.
2. Use of the ^238^U^1^H/^238^U and ^238^U^16^O^1^H/^238^U^16^O ratios to correct for the molecular hydride interferences of ^235^U^1^H on ^236^U and ^235^U^16^O^1^H on ^236^U^16^O does not uniformly improve the ^236^U/^238^U or ^236^U^16^O/^238^U^16^O ratio determination. The lack of uniform improvement appears to be related to the fact that the ^238^U^1^H/^238^U and ^238^U^16^O^1^H/^238^U^16^O ratios tend to behave consistently (i.e. they either decrease, remain stable, or increase) within the individual analyses whereas the ^236^U/^238^U and ^236^U^16^O/^238^U1^6^O are considerably more scattered. When combined with the fact that the comparatively large signals for ^238^U^1^H and ^238^U^16^O^1^H must be applied to the considerably lower ^236^U and ^236^U^16^O signals by way of the ^235^U/^238^U or ^235^U^16^O/^238^U^16^O ratio, the result is a magnification in the amount of deviance exhibited by the corrected ^236^U/^238^U and ^236^U^16^O/^238^U^16^O ratios.
3. Examination of the relationships between the various elemental, molecular oxide, and molecular hydride secondary ion ratios suggest that it may be eventually possible to model hydride (both the ^235^U^1^H and ^235^U^16^O^1^H) formation rates as a function of the non-hydride molecular oxide production rates taking place within a particular analysis. This conclusion is based on the existence of co-variance between the molecular hydride and oxide production rates. However, more work is needed to further assess this possibility.

## Methods

### Description of materials and mounting techniques

The UO_2_ analyzed in this study was produced by calcination of UO_3_ (produced via internal gelation as described in^9^) spheres at 600 °C for 5 h followed by sintering at 1,700 °C for 3 h in a reducing atmosphere (Ar w/4% H_2_). A large microsphere (≥ 500 µm diameter) of this sintered material was randomly selected from the batch and coarsely crushed before being crudely dispersed onto the various substrates utilized for NanoSIMS analysis by using a pair of stainless-steel tweezers. A small shard of the crushed material was also routed for uranium isotopic measurements via solution multi collector-inductively coupled plasma-mass spectrometry (MC-ICP-MS) which will be described in the subsequent paragraph. For NanoSIMS analysis, the substrates consisted of a polished carbon planchet (Ted Pella, Inc), a silicon wafer (Nova Electronic Materials), high purity (> 99.99%) platinum foil (Aldrich), a polished aluminum billet, and a carbon sticky tab (Ted Pella, Inc). Each of the substrates was observed to produce a ^238^U secondary ion signal ≤ than the detector background of 0.01 c/s (averaged over 5 min). Each of the substrates (except the sticky tab) were prepared to receive the UO_2_ in two ways. One way was completely bare such that the particles were only adhered to surface electrostatically. The other way was with a thin coating of Apiezon-L grease. The grease coating was applied by smearing a small quantity of it onto the substrate followed by use of a cotton cleanroom wipe to smoothen and remove the bulk of the grease such that only a thin veneer of grease remained on the substrate.

In addition to the crushed UO_2_ mounted on the various greased and ungreased substrates, a cross-sectioned and polished sphere taken from the same batch was also analyzed. This sphere was mounted in Buehler Epothin2 epoxy followed by use of silicon-carbide and diamond based abrasives (down to ¼ µm grit) to produce a flat surface that was then coated in 50 nm of Au using a Cressington 208 HR sputter coater outfitted with an MTM-20 thickness controller prior to analysis on the NanoSIMS 50L. A shard of the NIST-610 glass reference material was also analyzed. It was prepared in the same way as the cross-sectioned and polished UO2. The U isotopic composition of the NIST-610 glass, determined via multiple techniques, has previously been reported by^7^ as follows: ^234^U/^238^U = 0.00000945(5), ^235^U/^238^U = 0.0023856(7), and ^236^U/^238^U = 0.00004314(4).

### NanoSIMS analysis

The various sample formats (greased and ungreased substrates containing crushed UO_2_ as well as the polished UO_2_ and NIST-610 glass) were all analyzed using a NanoSIMS 50L (described in^10^) at Oak Ridge National Laboratory in August of 2019. The instrument used in this study was equipped with the Hyperion-II radio-frequency plasma oxygen ion source (described in^11^). For the U isotopic measurements, the NanoSIMS primary column was tuned through use of the L1 and L0 lenses and D1 aperture to achieve a ~ 1 µm diameter 200 pA beam of O− ions on the sample surface. The exception to this beam current is for analyses on the NIST-610 glass, which only contains ppm levels of U. For these analyses, the L1 and L0 lenses were tuned to achieve a 2 nA beam. The NanoSIMS 50L entrance and aperture slits were tuned, in conjunction with the quadrupole lens, to achieve a mass resolving power of ~ 7,000 (m/∆m) at ~ 40% relative transmission. While the NanoSIMS 50L used in this study is equipped with seven moveable detector positions (each one consisting of an inter-changeable electron multiplier and Faraday cup), mass dispersion is insufficient at the mass ranges used in this study to be able to analyze the various U isotopes within the same magnetic field. Therefore, individual analyses were conducted using a peak hoping approach with the detector and magnetic field configuration outlined in Table 1. The secondary ion imaging capability was utilized to identify the locations where individual analyses were performed within a broader region of interest (typically 50 × 50 µm area). Following a minor tuning of the secondary ion extraction and steering optics to account for slight topographical variations between analysis positions, data were collected as the primary beam was scanned over a 5 × 5 µm area using the Cameca ‘isotopes’ acquisition mode for twenty cycles (each cycle lasting 6.5 s; total analysis time ~ ≈ 70 min including magnet cycling and settling times). Prior to starting the analyses reported in this study, detector noise levels were observed to be within the instrument’s factory specifications (< 0.01 c/s averaged over five minutes). The data exported from the NanoSIMS for processing (described in “Discussion”) was in the form of counts/cycle and was uncorrected for detector deadtime.

### Solution MC-ICP-MS

As mentioned in “Use of the ^236^U^16^O/^238^U^16^O vs the ^236^U/^238^U ratio”, an aliquot (consisting of a single shard) of the crushed UO_2_ was also analyzed via MC-IPC-MS to obtain its uranium isotopic composition. The UO_2_ was digested in 0.5 mL of 4 M HNO_3_ for ~ 2 weeks at ambient conditions before analysis on a Thermo Scientific NeptunePlus MC-IPC-MS equipped with a jet interface and nuclear package. During analysis ^234^U, ^235^U, ^236^U, and ^238^U were placed on adjacent Faraday cups connected to 10^11^ and 10^13^ Ω electronically calibrated amplifiers using a 0.3 gain calibration card. The tau factor as well as a 20-min Faraday cup baseline measurement were performed. Sample solutions were introduced with a ~ 52 µL min^−1^ Elemental Scientific Inc. integrated PFA nebulizer into a quartz Elemental Scientific Inc. Apex Omega. The instrument sensitivity was ~ 1.4 V of signal per nanogram of uranium. The UO_2_ sample was analyzed in conjunction with other unknown samples (all having depleted ^235^U/^238^U isotopic compositions) using a standard sample bracketing method with 2% HNO_3_ washout blanks placed before each standard/sample. Mass fraction corrections were determined with New Brunswick Laboratory Program Office Certified Reference Material U010. Unknown solutions were corrected for mass fractionation, instrument blank, baseline, and gain using established protocols in the Nuclear Analytical Chemistry and Isotopics Laboratory at Oak Ridge National Laboratory. Controls of IRMM 183, IRMM 184, and Oak Ridge National Laboratory WRM were analyzed throughout the sequence to monitor instrument operation. Final calculated isotopic values were provided with Guide to Uncertainty in Measurement^12^ compliant uncertainties. This resulted in the following isotopic ratios and 2σ uncertainties for the UO_2_: ^234^U/^238^U = 0.0000086(2), ^235^U/^238^U = 0.002153(1), ^236^U/^238^U = 0.00002576(4).

## Supplementary information

Supplementary Information 1.
